# Development of Oral Tablets of Nebivolol with Improved Dissolution Properties, Based on Its Combinations with Cyclodextrins

**DOI:** 10.3390/pharmaceutics16050633

**Published:** 2024-05-09

**Authors:** Francesca Maestrelli, Marzia Cirri, Natascia Mennini, Silvia Fiani, Beatrice Stoppacciaro, Paola Mura

**Affiliations:** Department of Chemistry, School of Human Health Sciences, University of Florence, Via Ugo Schiff 6, 50019 Florence, Italy; francesca.maestrelli@unifi.it (F.M.); natascia.mennini@unifi.it (N.M.); silvia.fiani@unifi.it (S.F.);

**Keywords:** nebivolol, sulfobutylether-βCyclodextrin, coevaporation, oral tablets, dissolution rate

## Abstract

New oral tablets of nebivolol have been developed aiming to improve, by cyclodextrin (CD) complexation, its low solubility/dissolution properties—the main reason behind its poor/variable oral bioavailability. Phase-solubility studies, performed using βCD and highly-soluble βCD-derivatives, indicated sulfobutylether-βCD (SBEβCD) as the best solubilizing/complexing agent. Solid drug-SBEβCD systems were prepared by different methods and characterized for solid-state and dissolution properties. The coevaporated product was chosen for tablet development since it provided the highest dissolution rate (100% increase in dissolved drug at 10 min) and almost complete drug amorphization/complexation. The developed tablets reached the goal, allowing us to achieve 100% dissolved drug at 60 min, compared to 66% and 64% obtained, respectively, with a reference tablet without CD and a commercial tablet. However, the percentage dissolved after 10 min from such tablets was only 10% higher than the reference. This was ascribed to the potential binding/compacting abilities of SBEβCD, reflected in the greater hardness and longer disintegration times of the new tablets than the reference (7.64 vs. 1.06 min). A capsule formulation with the same composition of nebivolol-SBEβCD tablets showed about a 90% increase in dissolved drug after 5 min compared to the reference tablet, and reached 100% dissolved drug after only 20 min.

## 1. Introduction

Hypertension is considered as a major public health concern, being one of the leading causes of morbidity and mortality in the current world population [1]. The effective control of blood pressure is fundamental in hypertension management, to decrease the risks of hypertension-related pathologies and death [2]. Nebivolol hydrochloride (NEB) is a third-generation highly-selective β1-adrenergic receptor antagonist particularly recommended in the treatment of hypertension [3,4,5,6] and it is available on the market as tablets. Beneficial effects of NEB in the prevention and treatment of diabetic neuropathy have also been reported [7]. NEB has been classified as a Class II drug according to the biopharmaceutical classification system (BCS), and it is characterized by a high membrane permeability but a poor solubility and dissolution rate. Unfortunately, despite its favorable partition coefficient value, it presents problems of poor and variable oral bioavailability, mainly related to its low aqueous solubility [5,8].

A variety of formulation approaches has been explored to improve NEB solubility, and then enhance its clinical efficacy, including the development of solid dispersions with hydrophilic polymers [9,10] nanosuspension tablets [11], immediate-release tablets [12], nanofibrous sheets [13], solidified self-nanoemulsions [14], cocrystals [15], microemulsions [16] and liquisolid compacts [17]. Cyclodextrin (CD) complexation is another method widely applied to enhance the solubility and bioavailability of poorly soluble drugs [18,19]. CDs are cyclic oligosaccharides characterized by their particular structure composed by an internal hydrophobic cavity and an external hydrophilic surface, enabling them to form stable complexes with a variety of hydrophobic molecules [20]. Natural and chemically modified CDs have been largely used in the pharmaceutical field in virtue of their ability to form inclusion complexes with hydrophobic drug molecules, improving their water solubility, stability and bioavailability and decreasing their side effects [21,22]. However, the effectiveness of such a strategy towards NEB has been very scarcely explored to date [23,24], and the design and development of dosage forms containing NEB as CD complex has never yet been investigated. On the contrary, also considering its low daily dosage, NEB appears as an ideal candidate for such a strategy, which is also applicable to conventional dosage forms, without requiring the development of appropriate delivery systems, thus further being particularly attractive to reduce time and production costs. 

Therefore, the purpose of this study was to exploit CD complexation to develop more effective conventional oral tablets of NEB, endowed with improved drug dissolution properties. With this aim, an initial screening based on phase-solubility studies was performed to evaluate the solubilizing and complexing power towards the drug of native βCD and some highly-soluble βCD-derivatives, in order to select the most effective partner for NEB. Native βCD was selected, since, despite the limits due to its relatively low water solubility, it is until now the most largely used CD, its cavity being suitable for accommodating a large variety of drugs. As for the hydrosoluble βCD-derivatives, methyl-βCD was selected for its highest aqueous solubility, while hydroxypropyl-βCD and particularly sulfobutylether-βCD were chosen considering not only their good water solubility, but also their complete absence of toxicity, being the only βCD-derivatives also allowed for parenteral use [25,26].

Solid drug–CD binary systems were then prepared with the selected CDs, evaluating and comparing the efficacy of different preparation methods (grinding, kneading and coevaporation). The solid-state features of the various binary systems were investigated by differential scanning calorimetry and X-ray powder diffraction analyses and their dissolution performance was compared to that of the simple physical mixtures and the pure drug. The best product was finally selected for the development of conventional tablets, which were characterized for technological properties according to the Eur. Pharmacopoeia tests, and tested for dissolution properties in comparison with an NEB commercial tablet.

## 2. Materials and Methods

### 2.1. Materials

Nebivolol HCl (NEB) (2,2′-Azanediylbis(1-(6-fluorochroman-2-yl) ethanol) hydrochloride) was a kind gift from Menarini S.p.a. (L’Aquila, Italy). Beta-cyclodextrin (βCD) was from Sigma (St. Louis, MO, USA). Hydroxypropyl-βCD (HPβCD, average substitution degree 0.62) was kindly supplied by Roquette (Lestrem, France), and sulfobutylether-βCD (Dexolve^®^) (SBEβCD, average substitution degree 6.5) was a kind gift from Cyclolab Ltd. (Budapest, Hungary). Randomly substituted methyl-βCD (RAMEB, average substitution degree 1.8) was from Wacker Chemie (München, Germany). Explotab^®^ (sodium starch glycolate) was from JRS Pharma (Rosenberg, Germany), and Mg stearate, polyvinylpyrrolidone (PVP) K90, mannitol and CaHPO_4_ were from Sigma (St. Louis, MO, USA). All other chemicals and solvents were of analytical reagent grade.

### 2.2. Phase-Solubility Studies

Phase-solubility studies were performed at 37 °C by adding an excess amount of NEB to aliquots of 10 mL of simulated gastric medium (HCl 0.1 N pH 1.1, sodium glycine carbonate, sodium chloride) containing increasing CD amounts. The CD concentration range was 0–12.5 mM for βCD (due to its limited aqueous solubility) and 0–25 mM for all other tested CDs. The ampoules were closed and kept at 37 °C under magnetic stirring (750 rpm) until equilibrium (3 days). After that, a sample of solution was taken (Millipore filter syringe, pore size 0.45 µm) and spectrophotometrically analyzed at 281 nm (UV/Vis 1601 Shimadzu Spectrophotometer, Tokyo, Japan) for drug content determination. The dosage method has been validated following the ICH guidance Q2(R2) [27]. The linearity was established by using a seven-point calibration curve in a concentration ranging from 15 to 80 mg/L. The obtained linear regression equation was y = 0.0122x + 0.0005 (R^2^ = 0.999). The accuracy of the method was assessed by measuring three known concentrations, represented by the lowest, medium and highest values of the calibration curve, respectively. The precision was evaluated by dosing three drug concentration levels, performing three replications for each sample, in order to determine both repeatability and intermediate precision. The resulting LOQ and LOD values were 8.00 mg/L and 2.67 mg/L, respectively. It has been verified that the presence of CDs did not have any impact in the NEB spectrophotometric analysis. Studies were conducted in triplicate (coefficient of variation < 2.5%). 

The stability constants (*K*_1:1_) of the various complexes were obtained using the following equation [28]:

$$K_{1:1}=\frac{slope}{S_{0}*\left(1-slope\right)}$$

where *slope* is the slope of the phase-solubility profile and *S*_0_ the NEB solubility in the simulated gastric medium.

The complexation efficiency (*CE*) was calculated according to the following equation [29]:

$$CE=\frac{\left[NEB-CD\right]}{\left[CD\right]}=\frac{slope}{1-slope}$$

where [*NEB* − *CD*] and [*CD*] are the concentrations of dissolved complex and free *CD*, respectively, and *slope* is the slope of the phase-solubility profile.

### 2.3. Preparation of Drug–CD Solid Systems

Equimolar drug–CD solid systems were obtained by cogrinding, kneading and coevaporation. Simple physical mixtures (PMs) were also prepared, for comparison purposes, by 15 min of tumble-mixing equimolar amounts of the two components previously sieved (75–150 µm).

Coground products (GR) were prepared by ball-milling at 24 Hertz for 30 min of the PM in a high-energy vibrational micromill (MM200 Retsch GmbH, Haan, Germany. Kneaded products (KNs) were obtained by wetting the PM with small amounts of a 1:1 *v*/*v* ethanol/H_2_O mixture and then properly grinding the sludge with a pestle, until the solvent was fully removed; the obtained powder was kept for 24 h at 40 °C for achieving a complete drying. Coevaporated products (COE) were prepared by solubilizing NEB in the smallest volume of ethanol and CD in water; the solutions were then joined in a ground-stoppered flask and evaporated with rotavapor at 80 °C and 150 rpm; the resultant product was kept in an oven at 40 °C for 24 h. 

### 2.4. Solid-State Characterization of Drug–CD Binary Systems

Solid-state characterization of the individual components and of the different drug–CD binary systems was carried out by differential scanning calorimetry (DSC) and powder X-ray diffractometry (PXRD).

DSC analyses were performed with a Mettler TA4000 Star^e^ software (Version 9.00) system equipped with a DSC25 cell (Mettler-Toledo, Greifensee, Switzerland), by accurately weighing 5–10 mg samples (MX5 Microbalance, Mettler-Toledo, Greifensee, Switzerland) that were placed in pierced aluminum pans and scanned at a heating rate of 10 °C min^−1^ under a static air atmosphere, in the 30–300 °C temperature range. The instrument was calibrated with Indium as a standard (99.98% purity; melting point 156.61 °C; fusion enthalpy 28.71 J/g). Measurements were performed in triplicate. 

PXRD patterns were recorded with a theta–theta Bruker D8-Advance apparatus (Billerica, MA, USA) with Cu Kα radiation and a graphite monochromator, at 40 mV voltage and 55 mA current. All samples were analyzed at room temperature in the 5–40° 2θ range, at a scan rate of 0.05°/s. 

### 2.5. Dissolution Studies

Dissolution tests were performed according to the dispersed amount method [30], which is commonly used at the level of pre-formulation studies [31,32,33]. Briefly, samples containing 180 mg of drug (as such or as 1:1 mol/mol binary system with CDs) were added in a 150 mL vessel containing 75 mL of the pH 1.1 simulated gastric medium, thermostated at 37 ± 0.5 °C; the system was kept under stirring using a shaker in glass, endowed with three blades (19 mm diameter) rotating at 100 rpm, which was immersed in the center of the vessel at 25 mm from the bottom. At predefined times, aliquots (3 mL) were collected, filtered (Millipore membrane filter, pore size 0.45 µm), replaced with an equal volume of fresh medium and UV-dosed as in the previous Section 2.2. A correction to account for the dissolution medium dilution was made by applying the following formula:

Ci_corr_ = Ci + (Vp/V_0_) × Ci

where Ci_corr_ is the corrected concentration, Vp the withdrawal volume, V_0_ the total volume of the dissolution medium and Ci the read concentration. 

The results are the mean of four experiments (coefficient of variation < 5%). NEB dissolution performance was evaluated by considering the percentage of NEB dissolved at 10 min, as indicative of its dissolution rate, and the dissolution efficiency at the conclusion of the experiment (60 min), as indicative of the efficiency of the entire process. The dissolution efficiency (D.E.) was estimated from the area under the dissolution curve at time t (measured using the trapezoidal rule) and expressed as the percentage of the area of the rectangle described by 100% dissolution in the same time [32]. All the data have been analyzed by ANOVA (one-way analysis of variance) (GraphPad Prism version 4.00, GraphPad Software, San Diego, CA, USA). Differences have been considered statistically significant when *p* < 0.05.

### 2.6. Preparation and Characterization of Tablets

Tablets containing 10.9 mg of drug as a hydrochloride salt (equivalent to 10 mg of drug), as received or as an equimolar combination with the selected CD, were obtained by direct compression. PVP K90 (50 mg) was used as a binder, Explotab^®^ (sodium starch glycolate) (50 mg) as a super-disintegrant, mannitol (100 mg) and Calcium hydrogen phosphate (100 mg) as fillers-diluents and Mg stearate (1% *w*/*w*) as a lubricant. To maintain the tablet final weight as constant, in the case of tablets containing the CD, the amount of mannitol was adequately reduced. The powders were accurately weighed and then mixed in a turbula mixer for 15 min; after that, the mixture was compressed using a hydraulic press at 2.5 tons for 3 min. The tablets were characterized for weight uniformity, hardness, friability and disintegration time, all performed according to the FU XII and Ph. Eur. 11th Ed. official tests and compared with a marketed formulation (Nebivololo Mylan Italia, Mylan S.p.A., Milan, Italy). 

Weight uniformity: The mean weight of twenty randomly selected tablets, individually weighed using an electronic precision balance (Radwag mod AS 220.X2, Bioclass, Pistoia, Italy) was determined; the percentage weight variation of the individual tablets from the mean weight was then calculated. 

The hardness of the tablets (kg/cm^2^, mean of ten measurements) was determined using a Monsanto hardness tester. 

A friability tester (Erweka GmbH, Langen, Germany) was used for the friability evaluation. Twenty tablets randomly selected from each batch were accurately weighed and placed in the apparatus revolving at 25 rpm. After 4 min, the tablets were dedusted and reweighed and the percentage loss in weight was determined. 

The disintegration test was performed by individually placing six tablets, randomly selected from each batch, in each tube of the specific FU XII apparatus (Tecnogalenica, Labnova srl, Milan, Italy), containing the pH 1.1 solution simulating the gastric medium, thermostated at 37 °C; the time needed for the complete disintegration of all tablets has been determined.

The dissolution test was instead carried out in the same conditions previously used for the drug and the drug–CD binary systems (see par. 2.5) in order to be able to compare the results and evaluate the effect of the formulation and tableting on the drug dissolution behavior. It was preliminarily checked that the presence of the excipients did not give rise to interferences in the UV dosage of NEB. Experiments were repeated three times (CV < 4.0%).

### 2.7. Compatibility Studies

Compatibility studies between the drug and the selected excipients have been performed by DSC analysis, in order to verify the absence of solid-state interactions that could affect the drug stability and/or bioavailability and then its safety and/or clinical efficacy. With this aim, the DSC curves of the drug and each examined excipient were compared with those of their 1:1 *w*/*w* physical mixture. This *w*/*w* ratio was selected to maximize the possibility of detecting any potential interaction between the components. DSC analyses were performed with a Mettler TA4000 Star^e^ software system equipped with a DSC25 cell (Mettler-Toledo, Greifensee, Switzerland), under the same experimental conditions described above (see par. 2.4).

## 3. Results

### 3.1. Phase-Solubility Studies

**Figure 1:**
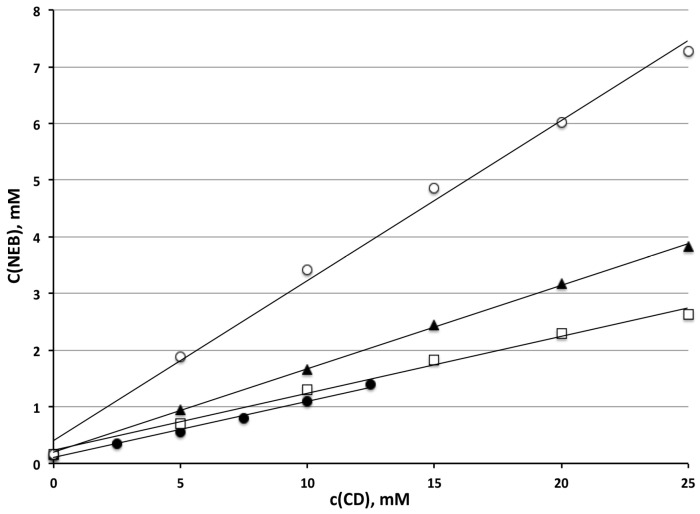
Phase-solubility diagrams of NEB in the presence of βCD (●), HPβCD (◻), RAMEB (▲) and SBEβCD (○) at 37 °C in simulated gastric medium (pH 1.1).

The results of phase-solubility studies performed in simulated gastric medium (pH 1.1) in the presence of increasing amounts of βCD and some highly soluble βCD-derivatives, are shown in Figure 1 and summarized in Table 1, in terms of the stability constants of the complexes, complexation efficiency and solubilizing efficiency values. 

As can be seen, in all cases a linear increase in NEB solubility was found with the increasing CD concentration. This behavior is typical of A_L_-type diagrams and considered indicative of the formation of soluble complexes with a 1:1 mol:mol host–guest stoichiometry [28]. As expected, βCD showed the lowest solubilizing efficiency, due to its limited water solubility. However, unexpectedly, the complexing ability of HPβCD was very similar to that of the native CD, despite its much higher water solubility due to the presence of the hydroxypropyl substituents. On the contrary, a clearly better performance of RAMEB and even more so of SBEβCD was evident.

### 3.2. Preparation and Characterization of Drug–CD Solid Systems

Considering the proven influence that the preparation method of the drug–CD systems can have on their physicochemical properties and then on their final performance [34,35,36], solid binary systems of NEB with the examined CDs were prepared by different methods, i.e., kneading (KN), cogrinding (GR) and coevaporation COE), in order to select the most effective technique by taking as a reference the corresponding simple physical mixtures (PMs). All the systems were prepared at a 1:1 molar ratio, as indicated by phase-solubility studies. The obtained products were then characterized for solid-state properties by DSC and XRPD analyses and investigated for dissolution rate behavior.

#### 3.2.1. Solid-State Studies

**Figure 2:**
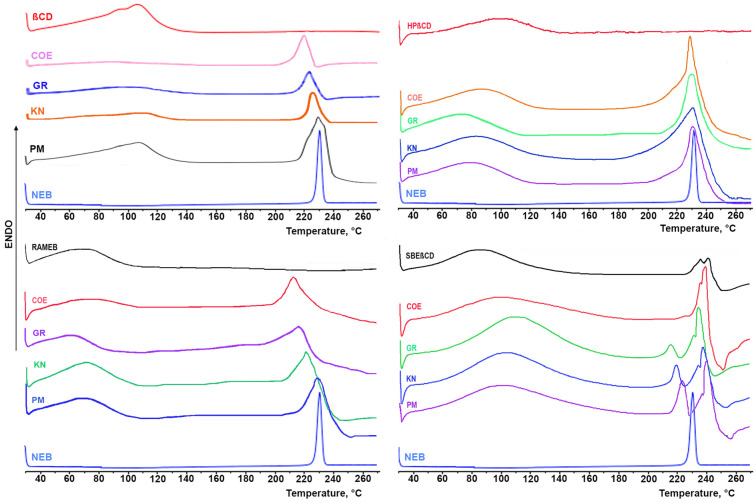
DSC curves of pure drug and CDs, and of their respective 1:1 mol:mol physical mixtures (PMs), kneaded (KN), coground (GR) and coevaporated (COE) products.

The DSC curves of the series of drug products with crystalline native βCD and amorphous βCD-derivatives are shown in Figure 2, together with those of the respective pure components.

The thermal curve of NEB was typical of a crystalline, anhydrous pure substance, exhibiting only a sharp and intense endothermic peak at 230.7 °C, due to the drug melting process. It was verified that the thermal behavior of the drug alone, subjected to the same treatments used for preparing its binary systems with the CDs, remained in all cases almost unmodified. The DSC curves of all the examined CDs were characterized, in the examined temperature range, by a broad endothermic band from 40 to 120 °C, due to their dehydration; moreover, SBEβCD presented an additional irregular and broad endothermic effect in the range between 250 and 270 °C attributed to decomposition phenomena. The drug endothermic peak was still evident in the DSC curves of all the PMs of the drug with the examined CDs, exhibiting only minor changes in melting peak and fusion enthalpy values, attributable to the simple blending with the CD and indicative, as expected, of the absence of drug–CD solid-state interactions. However, unfortunately, the results of further DSC studies were of limited usefulness in choosing the preparation techniques most powerful in inducing effective solid-state interactions between the components. In fact, the drug endothermic band was also observed in all of the series of the different products with the various CDs, only showing a more or less evident broadening and/or shift to a lower temperature, depending on both the CD type and the preparation method used, suggesting only partial drug interaction/amorphization. Interestingly, a different behavior was observed only in in the case of the NEB COE systems with SBEβCD where the drug melting endotherm, well detectable in the other series of products with this CD, right before its decomposition band, completely disappeared, indicating full drug complexation and/or amorphization. Thus, based on these findings, in the case of NEB–SBEβCD, coevaporation was revealed as the most effective technique in inducing drug–CD solid-state interactions.

**Figure 3:**
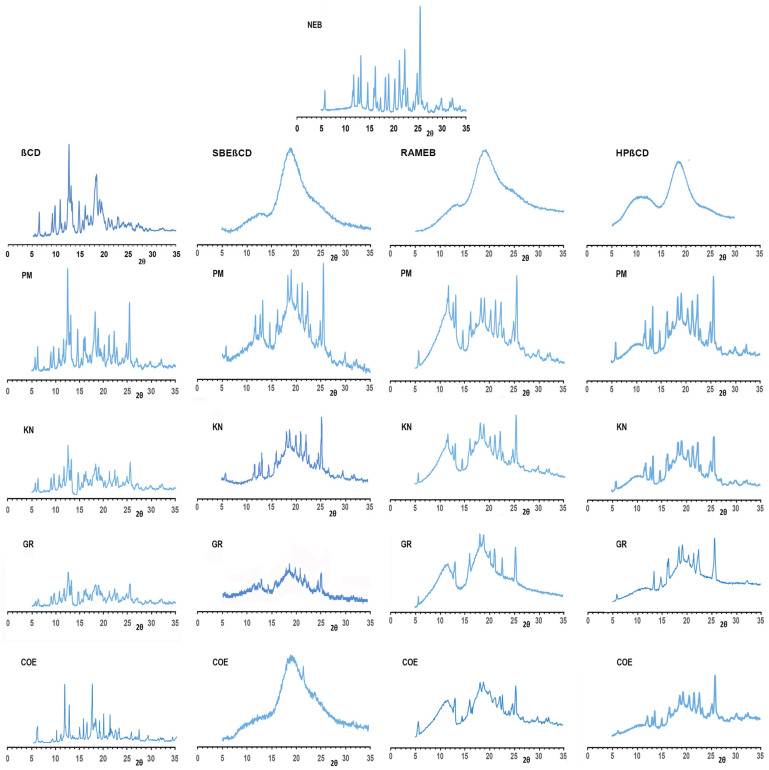
PXRD spectra of pure drug and CDs, and of their equimolar physical mixtures (PMs), kneaded (KN), coground (GR) and coevaporated (COE) products.

PXRD spectra of the different drug products with crystalline native βCD and amorphous βCD-derivatives, together with those of the respective pure components, are shown in Figure 3.

As can be seen, the patterns of NEB and native βCD were typical of crystalline products, exhibiting several characteristic sharp diffraction peaks. On the contrary, the PXRD spectra of all of the examined βCD-derivatives showed the halo pattern typical of amorphous substances. As expected, the spectra of the physical mixtures resulted in both cases in the simple superimposition of those of pure components, thus confirming the absence of solid-state interactions between the components. However, many of the characteristic diffraction peaks of the drug also remained well detectable in the various binary products, even though clearly being reduced in number and/or intensity, thus indicating only a partial drug–CD interaction. The only exception was observed in the case of the NEB–SBEβCD COE system, where an almost total NEB complexation and/or amorphization was obtained, as indicated by the nearly complete disappearance of its diffraction peaks. These results were in good agreement with those of DSC analysis and definitely confirmed SBEβCD as the most effective CD, as well as coevaporation as the most effective preparation technique of the complex in the solid state.

#### 3.2.2. Dissolution Rate Studies

Based on the results of both solid-state and phase-solubility studies, SBEβCD emerged as the most effective CD in interacting with the drug and increasing its water solubility, and then it was selected for dissolution rate studies, together with native βCD for comparison purposes.

**Figure 4:**
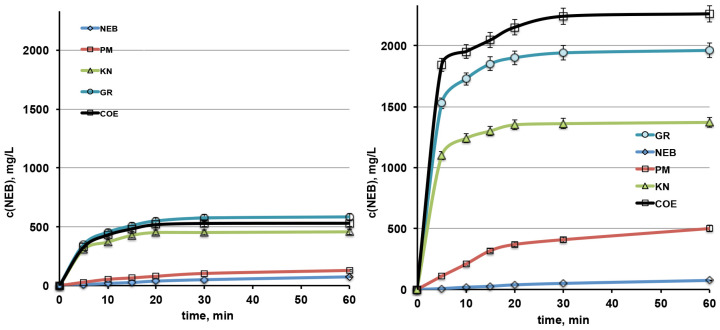
Dissolution rate profiles of NEB as received or as a 1:1 mol:mol physical mixture (PM), kneaded (KN), coground (GR) and coevaporated (COE) products with βCD (**left**) and SBEβCD (**right**).

The mean dissolution profiles of NEB from the various binary systems with the selected CDs are shown in Figure 4, while the results, in terms of the percentage dissolved at 10 and 30 min and the dissolution efficiency at 60 min, are presented in Table 2.

In the case of the drug systems with βCD, the PM gave rise only to a slight improvement in the NEB dissolution rate, mostly ascribable to the CD wetting properties, even though an almost immediate partial formation of the complex, upon contact with water, may also be considered. Better results were instead obtained with all of the other interaction products, which showed a rather comparable behavior, with an increase of up to 23 times of the drug amount dissolved after 10 min. 

A clearly better performance was shown by the NEB systems with SBEβCD, where the simple PM exhibited a dissolution behavior only slightly worse than those of the various interaction products with βCD. Moreover, further marked improvements in the drug dissolution rate were observed for KN, GR and especially COE products that gave rise, respectively, to increases of about 64, 90 and 102 times in the drug amount dissolved after 10 min, and 29, 41 and 48 times in DE at 60 min. Thus, these results further supported the choice of the COE with SBEβCD as the best product for the preparation of NEB tablets.

### 3.3. Preparation and Characterization of NEB Tablets

Based on the previous experience of our research group [31,33] and on a preliminary pre-formulation study, the following excipients were selected for the preparation of NEB tablets by direct compression: mannitol and CaHPO_4_ as diluents-fillers commonly used for direct compression, PVP K90 as a binder, Explotab^®^ as a super-disintegrant and Mg stearate as a lubricant. The evaluation of solid-state interactions that can occur between drug and excipients, possibly affecting drug stability and bioavailability, represents a fundamental step in the development of solid dosage forms. Then, before proceeding with further studies, the compatibility of the selected excipients with the drug was assessed by DSC analysis. In fact, this technique has been recognized as very effective for performing a rapid compatibility screening, allowing the fast detection of solid-state interactions, as well as changes in polymorphic forms or the conversion from crystalline to amorphous forms, with the further advantage of requiring only small amounts of samples [37,38]. With this aim, 1:1 *w*/*w* physical mixtures of NEB with each excipient were prepared and their thermal curves were compared with those of the respective pure components. It is assumed that, in the absence of interactions, the DSC curves of the blends are practically the sum of those of the single components [37]; on the contrary, the disappearance or shift of the drug melting peak, as well as the appearance of additional thermal events, is considered indicative of possible incompatibilities [39].

**Figure 5:**
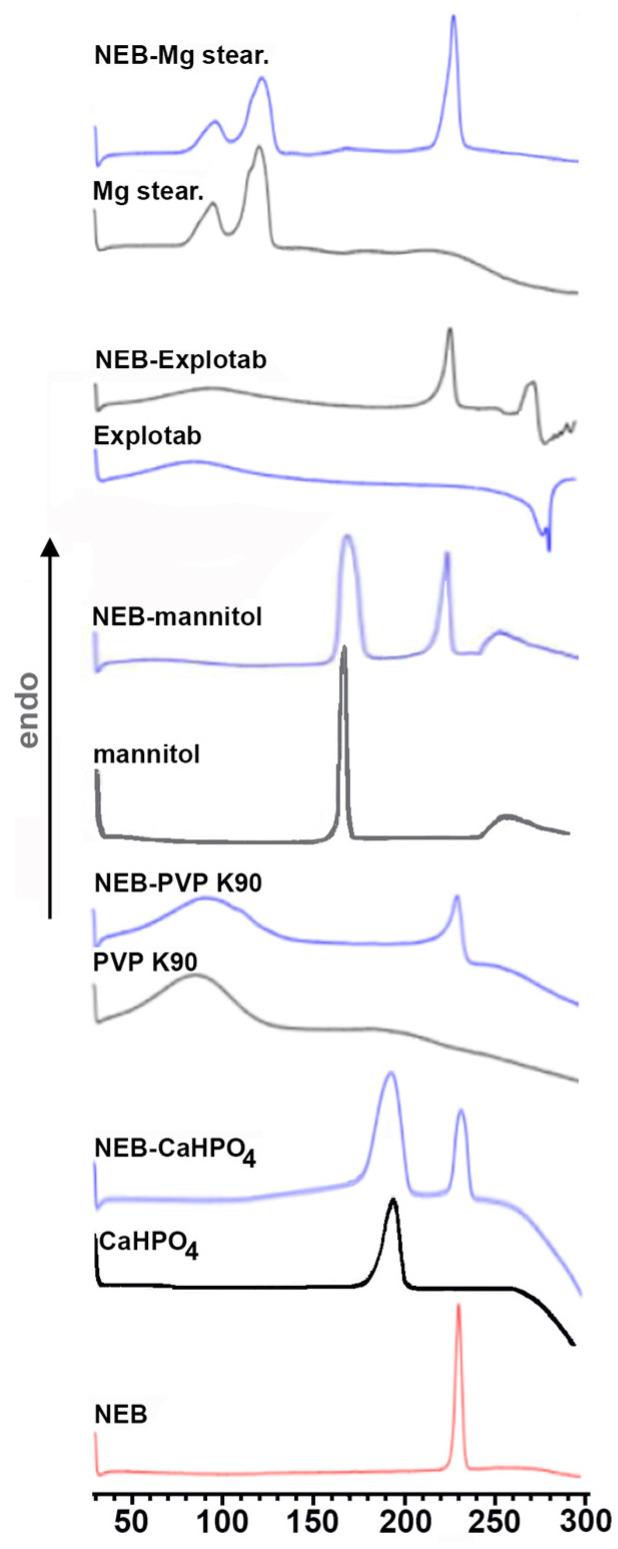
DSC curves of pure NEB and of the various tablet excipients (PVP K90, CaHPO_4_, Explotab^®^, mannitol and Mg stearate) and of their respective 1:1 *w*/*w* physical mixtures.

As can be seen in Figure 5, the typical melting endotherm of the drug was observed almost unchanged in the DSC curves of all of its physical mixtures with the different excipients, which resulted in the substantial superimposition of the DSC curves of their respective pure components. The observed reduction in the intensity of the drug melting peak is compatible with its 1:1 *w*/*w* blending with the excipients, particularly with the amorphous ones. These results indicated the absence of solid-state interactions between the components and, consequently, proved the compatibility with the drug of all the examined excipients.

Direct-compression tablets were then prepared containing 10 mg drug (as received or as PM or COE with SBEβCD), mannitol (100 mg), CaHPO_4_ (100 mg), PVP K90 (50 mg), Explotab^®^ (50 mg) and 1% *w*/*w* Mg stearate. The content of the diluent mannitol was suitably reduced in the formulations containing SBEβCD, so as to keep the final total weight of the tablets constant. The main technological properties of the different tablets are collected in Table 3, in comparison with those of a marketed formulation.

All the examined tablets exhibited a good weight homogeneity, low friability (always clearly less than 1%) and suitable hardness values comparable to those of the commercial formulation, indicating that they can be processed and handled without particular problems. However, interestingly, tablets containing the drug as received showed a clearly shorter disintegration time with respect to those containing its PM or COE systems with SBEβCD, thus suggesting some binder properties of SBEβCD that slowed down the disintegration process. The binder ability of βCD and of CD polymers has been proved [40], and βCD and HPβCD have been proposed as filler-binder excipients for direct compression [41]. However, it was found that βCD increased the tablets hardness without increasing the disintegration time, since it also acted as a disintegrant agent [42,43]. To the best of our knowledge, no data about these properties have yet been reported in the case of SBEβCD.

**Figure 6:**
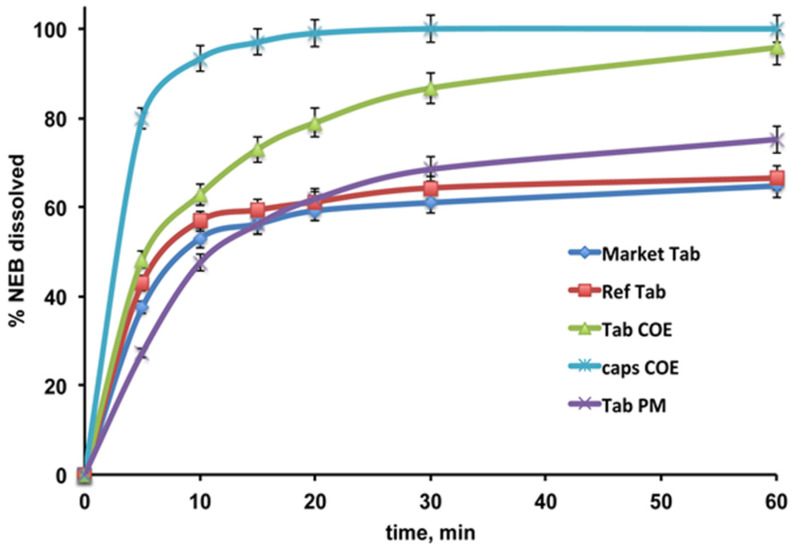
Dissolution profiles of NEB from the developed tablets containing the drug as received or as PM or COE with SBEβCD, from capsules with the same composition of the NEB-SBEβCD COE tablets, and from a marketed tablet formulation (Nebivololo Mylan Italia).

The dissolution profiles of NEB from the various tablets are shown in Figure 6.

As can be seen, the NEB dissolution profile from the reference tablet formulation, containing the drug as received, was rather similar to that from the commercial formulation, reaching only about 66 and 64% drug dissolved after 60 min, respectively. The presence of SBEβCD as a simple PM with the drug gave rise to only a limited increase in the % NEB dissolved at the end of the test (75%), and, unexpectedly, a lower dissolution rate was observed during the first 10 min with respect to the reference tablet. A clearly better dissolution profile was instead provided by the tablets containing the drug as COE with SBEβCD, which reached almost 100% dissolved drug after 60 min, and more than 80% dissolved after 30 min. However, in this case, the initial drug dissolution rate was also not as fast as expected, with the % dissolved after 10 min being only slightly higher than that from the reference tablet. This last finding could be a consequence of the observed increased hardness and disintegration times of both the tablets containing SBEβCD (see Table 3), which was attributed to the possible binding and compacting properties of this CD, as previously observed for other CDs, including βCD, HPβCD and polymeric CDs [40,41]. Then, in order to verify our hypothesis, a capsule formulation having the same composition of the NEB-SBEβCD COE tablets was prepared, so as to avoid the compression process. 

As can be seen in Figure 6, the capsule formulation actually showed a better NEB dissolution profile than the corresponding tablet, reaching 80% and 100% dissolved drug after only 5 and 20 min, respectively. Therefore, these results seemed to support our theory and could be explained with the absence of the formation of any compact solids retarding the dissolution, as a consequence of the compression force application. Thus, the capsule formulation enabled us to fully exploit the improved dissolution properties of the drug as an SBEβCD complex. 

## 4. Conclusions

The CD complexation of NEB proved to be an effective tool for improving its dissolution properties. In particular, among the various CDs tested, SBEβCD was revealed to be the best partner for the drug, showing the highest stability constant of the complex and the greatest complexing and solubilizing efficiency. Moreover, coevaporation emerged as the most effective preparation method of the complex in the solid state, being the only one able to give rise to the total NEB complexation and/or amorphization and allowing the largest increase in the drug dissolution rate, with an increase of more than 100% in the amount dissolved after 10 min, and an increase of about 50 times in dissolution efficiency at 60 min.

Tablets containing NEB as a coevaporated product with SBEβCD allowed us to reach 100% dissolved drug at 60 min, compared to 66% and 64% obtained, respectively, with the corresponding reference tablet formulation, containing the drug at the standard amount, and with a commercial tablet formulation. However, the increase in the percentage dissolved from the tablet formulations after 10 min was lower than that expected (only about 10% with respect to the reference formulation). This result could be ascribed to the potential binding and compacting abilities of SBEβCD, reflected in the longer disintegration times of the tablets containing the drug–CD complex than the reference tablet (7.64 vs. 1.06 min). In support of this hypothesis, a capsule formulation with the same composition of the NEB-SBEβCD COE tablets gave rise to an increase in dissolved drug after 5 min of about 90% with respect to the reference tablet and reached 100% dissolved drug after only 20 min.

In conclusion, on the basis of the obtained results, we can affirm that the proposed approach, based on NEB complexation with SBEβCD, was successful in achieving the desired goal, i.e., 100% dissolved drug from tablets at 60 min with no need to change the type of dosage form (and consequently not even the production plants), or add other excipients suitable for direct compression (and thus without problems of an increase in the weight and size of the tablets), due to the favorable technological properties of SBEβCD, thus resulting in a cost-saving strategy.

Further studies will be performed to investigate in more depth the reasons behind the unexpected increase in disintegration time observed in the case of the tablet formulation containing SBEβCD. We will explore how to overcome this issue by suitably modifying the tablet composition and/or the conditions of the compression process, possibly through an experimental design strategy or resorting to the artificial intelligence that has emerged as a powerful tool in reducing development time and costs [44].

## Figures and Tables

**Table 1:** Stability constants (*K*_1:1_), complexation efficiency (*CE*) and solubilizing efficiency of NEB complexes with the examined CDs.

*CD* Type	*K*_1:1_ (mM^−1^)	*CE*	Solubilizing Efficiency *
βCD	644	0.110	8.2
HPβCD	654	0.112	15.3
RAMEB	1011	0.173	22.4
SBEβCD	2306	0.394	42.6

* Ratio between solubility of drug in the presence of 25 mM *CD* (or 12.5 mM βCD and drug alone) in pH 1.1 simulated gastric medium at 37 °C.

**Table 2:** Percent dissolved at 10 (PD 10) and 30 (PD 30) min and dissolution efficiency at 60 min (DE 60) of NEB alone and from its physical mixtures (PMs), kneaded (KN), coground (GR) and coevaporated (COE) products with βCD and SBEβCD.

Sample	PD 10	PD 30	DE 60
NEB	0.8	2.1	1.8
NEB-βCD PM	2.2	4.3	3.7
NEB-βCD KN	15.4	18.8	17.2
NEB-βCD GR	18.9	23.9	21.5
NEB-βCD COE	17.9	22.0	19.9
NEB-SΒΕβCD PM	8.7	17.0	15.0
NEB-SΒΕβCD KN	51.6	56.7	52.8
NEB-SΒΕβCD GR	71.9	80.8	74.8
NEB-SΒΕβCD COE	81.3	93.3	86.1

**Table 3:** Technological properties of tablets containing NEB alone (reference tablet) or as a physical mixture (PM tablet) or coevaporated product (COE tablet) with SBEβCD, compared with a marketed formulation (Nebivololo Mylan Italia, Mylan S.p.A., Milan, Italy).

Tablet Sample	Weight (mg)	Disintegration Time (min)	Hardness (kg/cm^2^)	Friability (%)
Reference Tablet	314.6 ± 0.6	1.06 ± 0.29	7.75 ± 0.8	0.47
PM Tablet	314.2 ± 0.3	9.25 ± 1.50	11.9 ± 0.9	0.58
COE Tablet	314.1 ± 0.4	7.64 ± 1.38	11.5 ± 0.4	0.62
Nebivololo Mylan	232.3 ± 2.3	2.24 ± 0.23	7.33 ± 0.3	0.40

## Data Availability

Data are contained within the article.

## Abbreviations

NEB	Nebivolol HCl
CD	Cyclodextrin
SBEβCD	Sulfobutylether-βCD
HPβCD	Hydroxypropyl-βCD
RAMEB	Randomly substituted methyl-βCD
PM	Physical mixture
GR	Coground products
COE	Coevaporated products
KN	Kneaded products
CE	Complexation efficiency
DE	Dissolution efficiency
DSC	Differential scanning calorimetry
PXRD	Powder X-ray diffractometry
